# A Need for a Paradigm Shift in Healthy Nutrition Research

**DOI:** 10.3389/fnut.2022.881465

**Published:** 2022-04-19

**Authors:** Alberto Aleta, Furio Brighenti, Olivier Jolliet, Erik Meijaard, Raanan Shamir, Yamir Moreno, Mario Rasetti

**Affiliations:** ^1^ISI Foundation, Turin, Italy; ^2^Human Nutrition Unit, Food and Drug Department, University of Parma, Parma, Italy; ^3^Department of Environmental Health Sciences, School of Public Health, University of Michigan, Ann Arbor, MI, United States; ^4^Borneo Futures, Bandar Seri Begawan, Brunei; ^5^Durrell Institute of Conservation and Ecology, University of Kent, Canterbury, United Kingdom; ^6^Institute of Gastroenterology, Nutrition and Liver Diseases, Schneider Children's Medical Center and Lea and Arieh Pickel Chair for Pediatric Research, Sackler Faculty of Medicine, Tel Aviv University, Tel Aviv, Israel; ^7^Institute for Biocomputation and Physics of Complex Systems (BIFI), University of Zaragoza, Zaragoza, Spain; ^8^Department of Theoretical Physics, Faculty of Sciences, University of Zaragoza, Zaragoza, Spain

**Keywords:** nutrition, complex systems, digitalization, data science, health

## Abstract

Research in the field of sustainable and healthy nutrition is calling for the application of the latest advances in seemingly unrelated domains such as complex systems and network sciences on the one hand and big data and artificial intelligence on the other. This is because the confluence of these fields, whose methodologies have experienced explosive growth in the last few years, promises to solve some of the more challenging problems in sustainable and healthy nutrition, i.e., integrating food and behavioral-based dietary guidelines. Focusing here primarily on nutrition and health, we discuss what kind of methodological shift is needed to open current disciplinary borders to the methods, languages, and knowledge of the digital era and a system thinking approach. Specifically, we advocate for the adoption of interdisciplinary, complex-systems-based research to tackle the huge challenge of dealing with an evolving interdependent system in which there are multiple scales—from the metabolome to the population level—, heterogeneous and—more often than not— incomplete data, and population changes subject to many behavioral and environmental pressures. To illustrate the importance of this methodological innovation we focus on the consumption aspects of nutrition rather than production, but we recognize the importance of system-wide studies that involve both these components of nutrition. We round off the paper by outlining some specific research directions that would make it possible to find new correlations and, possibly, causal relationships across scales and to answer pressing questions in the area of sustainable and healthy nutrition.

## Complex Interactions and Confounding Factors

Nutritional science is largely based on observational evidence that is often subject to many confounding factors. Traditionally, analytical epidemiology, and especially cohort studies, have relied on exploring multiple time- and exposure-related associations, while having fundamental shortcomings when it comes to proving causation. This approach seeks to link nutrients and other substances in food with individuals' health and disease. Methodologically, subjects are selected at random from a population, categorized according to their exposure (for instance, dietary habits, known confounding factors, etc.), and their health status monitored over time. This “follow the individual” approach presents many known methodological problems associated with time, cost, the internal and external validity of the sample, uncertainty of exposure, and individual variability in physiological response. On top of these problems, nutrition research needs to tackle the challenge of combining with diets and health entirely new sets of highly variable biological and non-biological covariates, such as intestinal microbiota at the molecular scale and behavioral contagion at the population level. The large concomitant uncertainties affect how scientists and health authorities define and interpret recommendations and guidelines and how the industry is consequently pushed toward innovation and reformulation. This, in turn, influences the food system in terms of food quality, availability and preferences, possibly resulting in further biological and behavioral changes. It also affects the entire food production systems, whose economic and environmental impacts have been largely considered in separate disciplines. As an historical example of loss function (the cost of being wrong), we should remember how the cholesterol-saturated fat hypothesis, endorsed by health authorities worldwide during the mid-1990s, led to the widespread introduction from the 1960s through the 1980s of hydrogenated fats as a healthy vegetable substitute for butter, and compare it to the current efforts to remove trans fats from the food system (1, 2).

## Making Sense of Big Data

**Figure 1:**
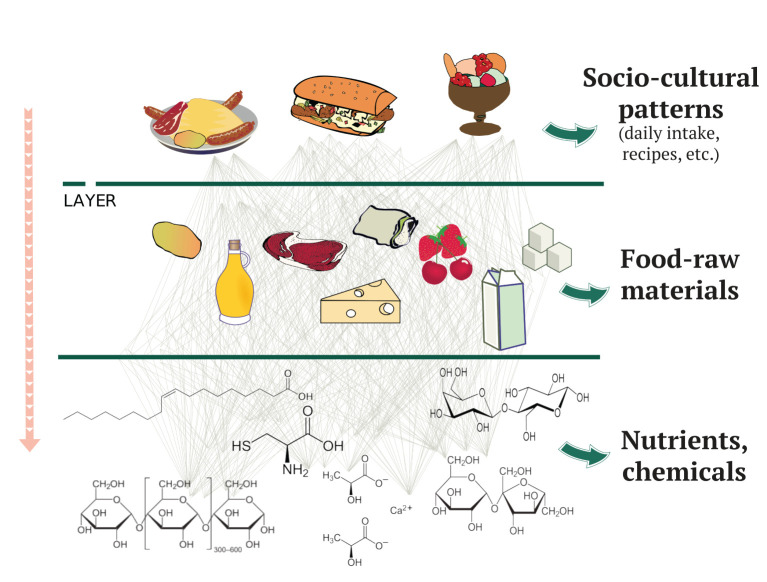
Multilayer schematic representation of the interactions that are involved at three different complexity levels. These interactions link together food composition and socio-cultural covariates. Each layer is represented as a network of interactions among the components at that scale.

We believe that the aforementioned methodological shortcomings and challenges represent an opportunity for a paradigm shift of increased application of techniques borrowed from other fields of science, particularly, from complexity science and artificial intelligence (AI). Certainly, unraveling causal relationships between nutrients, food ingredients, human health and disease implies the study of the “emergent” features generated by the complex, non-linear, multi-scale, and multi-level interactions and correlations among the several agents that make up such an interdependent system. A complexity-based approach places the focus on the connections among the components of the system, rather than on the single elements and looks for properties emerging from such associations. Figure 1 illustrates the proposed methodology. It schematically represents three scales of interactions: nutrients and chemicals, food raw materials, and finally the socio-cultural component of populations exemplified by diets, daily intake, and recipes. The mathematical tool that encodes and describes the interlinked relationships is a graph, which captures pairwise and other higher-order interactions, including situations in which the connections among the system components are best represented by a network of networks, namely, a multilayer network like the one depicted in Figure 1 (3). With these tools, it is possible to capture some of the non-trivial health impacts that food pairings might have, such as the pairing of red meat and garlic in the Mediterranean diet (4). Other higher-order systems, such as simplicial complexes, might be also used to capture further properties of these pairings as the changes produced during cooking, the non-trivial interactions among chemical compounds or their bioavailability. The proposed approach is also particularly suited to capture social covariates. Admittedly, at variance with communicable diseases, non-communicable diseases can spread by social influence, that is, there is mounting evidence that the prevalence of food-related diseases like obesity correlates with social habits, physical activity, behavioral factors, and the composition of an individual social circle (5–7). This needs to be incorporated in more traditional longitudinal analyses, where social factors are either disregarded or not methodologically formalized, with the result of inconsistencies across studies.

The advent of the digital era has revolutionized almost all fields of science, currently providing in a single year more data than what has been generated up to that moment by humankind. Nutrition is no exception, and the genomic era is uncovering an increasingly large number of metabolic networks of nutritional interest as well as an unprecedented quantity of data concerning the impact of nutrition on human health. Data on agriculture, food production and environmental impacts are also increasingly becoming available at global and local levels, providing unique opportunities to explore the complex interactions between a global production system, land use and deforestation, climate change, and local to global environmental impacts on humans and ecosystems. However, access to data is only the tip of the iceberg. There are many new challenges as we now need to be able to understand, interpret, and use this huge amount of data, both already available and to come. This is a task that can be successfully completed only by developing systems-based methodologies and artificial intelligence methods that fully enable cross-disciplinary integrative approaches and transform data into information and knowledge. A primary example of this systems-based approach is the creation of ontologies that connect knowledge across different disciplines related to nutrition research such as the Ontology for Nutritional Studies (ONS) (8). Additionally, big data analysis and artificial intelligence techniques can contribute to trace back behavioral traits in chemical consumption, including cross-comparison among geographical zones, inequality, income, historical, and other social determinants (9).

The combination of the methods and techniques from complex systems and artificial intelligence will enable the analysis of groups and clusters built across a large number of independent sets of data related to a large number of individuals—treated as a single multi-component ecological niche—to draw a picture able to explain the most likely interactions occurring in a country or continent-wide population. In other words, the possibility of linking nutrients to food to diet to health would allow for an “ecological” approach that could identify multiple relationships given by different aspects of the biological and social human environment out of the boundaries set for individual associations (10). These approaches also offer the possibilities to relate the nutritional effects of food consumption with the food production system, to also apprehend the socio-economic and environmental impacts of nutritional choices. This is key to understanding synergies and trade-offs, to then select or promote dietary changes and adapt the production system in ways that are beneficial for both human health and the environment. However, there are many difficulties inherent to following the proposed research methodology, ranging from the very existence of the data needed, to technical difficulties when it comes to glue together the different networks of interactions and their interdependencies. In what follows, we briefly describe in more detail two of these challenges.

## Need for a Data Standard

One key step is to characterize the exposure to the food system. To this end, the first and most pressing challenge is to obtain compositional data, which provide the building blocks of the interactions-based approach. In an ideal world, we should know in detail the chemical (nutrient and bioactive) composition of all foods and beverages that are available to consumers. The ingredients used for food preparation, additional characteristics such as energy density, sensorial attributes, type of processing, modes of production, trade patterns, and their socio-economic and environmental impacts (e.g., 247 indicators across 17 sustainable development goals), must also be known to draw a comprehensive picture of the existing food system. This might be explored through a detailed collection of data related to the existing food supply over a given space and time, taking advantage as much as possible of actual records provided by food producers, processors, and retailers. Nonetheless, one must be aware of the potential problems of the existing data. Classical, targeted analyses that look for a single or few compounds can be fooled and provide biased estimates of food composition (such as the substitution of protein by melamine in adulterated foods) or miss important chemical compounds that were not screened. Non-targeted analyses can alleviate some of these problems and have shown promising results in the detection of food fraud, but still cannot provide reliable enough quantitative estimates (11, 12). Furthermore, the analysis of real data availability reveals a situation that is far from the ideal scenario. Indeed, despite many efforts to gather, mine, and curate data, it is not homogeneous and often, it is noisy and incomplete. Several EU and US initiatives have tried to remedy this, such as EuroFIR, USDA or INFOODS, so far with a diverse degree of success (13, 14).

To exemplify the current problems associated with the quantity and quality of data for nutritional studies, we use as a typical case a common ingredient of the European—and particularly, Mediterranean—diet, namely, olive oil. The advantages of olive oil used to be attributed to its high oleic content, but nowadays it has been established that the small molecules composing the unsaponifiable fraction (representing 1–2% of the total oil weight) are also associated with beneficial health effects (15–17). However, olive oil composition is highly variable and depends on many genetic, environmental, and technological factors (18–21). This impacts both the fatty acid and unsaponifiable fraction compositions. For instance, in the case of fatty acids, cooler regions yield higher oleic acid than warmer climates. As such, the international olive oil council (IOOC) establishes wide ranges for its purity guidelines, with pure olive oil being composed of 55%−83% oleic acid (22).

**Figure 2:**
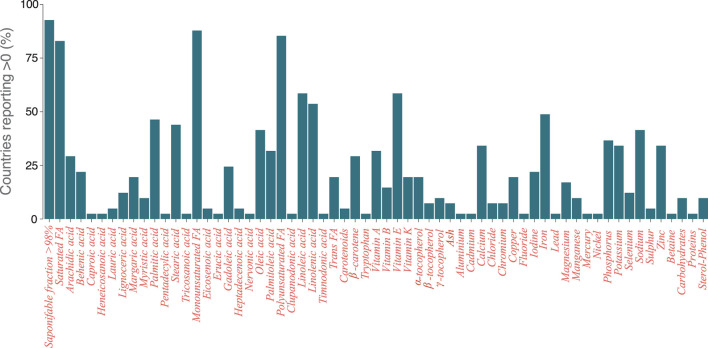
Percentage of countries whose food composition table quantifies each chemical compound for olive oil. We include as a quantified chemical those with an estimated quantity larger than 0, regardless of the specific amount or units shown in the database.

**Figure 3:**
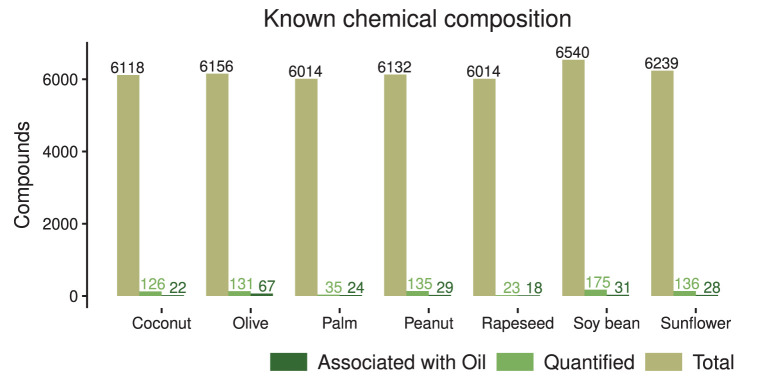
The number of chemical compounds associated with each source of vegetable oil in FooDB as of April 2020. From the total number of chemicals associated with each crop, the large majority are “expected [to be present] but not quantified”. The others can be associated with the ingredient in its raw form or in its oil. Most quantified compounds are associated with the raw product, and only a tiny fraction is explicitly associated with the oil. Note that for palm and rapeseed the amount of chemicals associated with the oil is almost the same as the quantified fraction, likely because these products are mostly consumed in their oil form.

Data incompleteness is also hampering progress in at least two ways, e.g., by yielding misleading results or unsubstantiated correlations (23, 24). A traditional nutritional approach would first reduce foodstuff to its main nutritional components using food composition databases. This is followed by monitoring nutrient intake by individuals, which is eventually linked statistically to outcomes, i.e., actual disease or, more often, markers of disease. This procedure however disregards non-trivial interactions that might arise between different food elements and neglects the effects of any chemical component not contained in the database. For instance, meta-analyses on the effect of monounsaturated fatty acids (MUFA) on coronary heart disease can yield inconsistent results if the source of the fat is not considered (25). Yet, if the source of MUFA is properly accounted for, it is observed that when it is of vegetable origin, the benefits are larger. As such, rather than promoting a low-fat intake, healthier sources of fat should be encouraged (26, 27). This implies that, either the specific chemical composition of those fatty acids, or other components that are contained in those products, can be partially responsible for the observed health effects. In other words, the whole complexity of food composition should be considered. This is nonetheless hardly achievable using currently available food composition databases. A review of the food composition tables of 74 countries looking for olive oil composition revealed that the information reported is usually scarce and inconsistent (Figure 2). Besides, several countries do not provide any information on olive oil, especially in countries in which this ingredient is uncommon. Interestingly, several databases report the presence of trans-unsaturated fatty acids, which should not be present in pure olive oil and can be associated with fraud and adulteration of the oil (22, 28). Furthermore, compounds not directly linked to nutrition are not reported. In addition, and of particular relevance is the fact that more general databases such as FooDB [one of the largest and most comprehensive resource on food constituents (29)] also show deep knowledge gaps in relation to the chemical composition of vegetable oils. For instance, of almost 6,000 chemical compounds associated with each oil crop from which oil is commonly extracted, roughly 100 are quantified, while the rest are “expected but not quantified” —which some authors refer to as nutritional dark-matter (4). Of those, <50 are tagged as associated with the oil. The others are associated with the raw element. This characterization is even worse in the case of palm and rapeseed oils, see Figure 3. The reason is that these products are mainly consumed in oil form and, thus, there are not many chemical compounds associated with their raw form in the database.

The preceding analysis clearly shows the need of increasing the quality and availability of food chemical composition data. Otherwise, the serious lack of data about the chemical composition of food raw material—here shown for vegetable oils—would make it hard to arrive to meaningful causal associations between such food and individuals' health and disease or to even compare benefits and inconveniences of different kinds of a given food material (e.g., vegetable oils). And, even if the composition was perfectly known, it would still be challenging to properly determine the effects of non-trivial interactions among chemical compounds, since as in any complex system the total is not just the addition of its parts. Another sensitive part of the data needed for nutritional studies is the variability associated with intake estimations. This is an area in which improving the techniques used to analyze nutrient intake is pressing and challenging but could capitalize on the proposed roadmap. The exposure assessment includes the amount of food eaten, but also meal occasions, hours of the day when meals are consumed, and other meal-related characteristics. This is a multidimensional problem as the actual human exposure to the food system strongly depends on exogenous factors such as individual economic capacity—workload and ability for accessing food stores and shops, food cost, etc.—, market share of different products, socio-cultural and geographical factors—local gastronomic habits linked to tradition and heritage—and, increasingly important, behavioral contagion and social pressure to adopt dietary habits—including traditional and social media, food labeling rules, ethical or religious constraints, etc. It is worth mentioning at this point that the same sort of difficulties arises when defining relations among nutrition and health status. Indeed, health is a multifactorial derivative that varies among individuals, time and location according to many metabolic, environmental and behavioral characteristics. Similarly, the production system, and its economic and environmental impacts over the entire local to global production supply chain would need to be much better characterized, with standardized data collection including food losses and combined with nutritional composition (30, 31). Therefore, providing recommendations to a global population about healthy diets from food systems regardless of cultural and behavioral covariates is a pressing issue whose satisfactory solution can only be obtained using a system-wide analysis. This is the second challenge that we discuss in more detail next.

## Revealing Interdependencies and Correlations in Food Consumption

**Figure 4:**
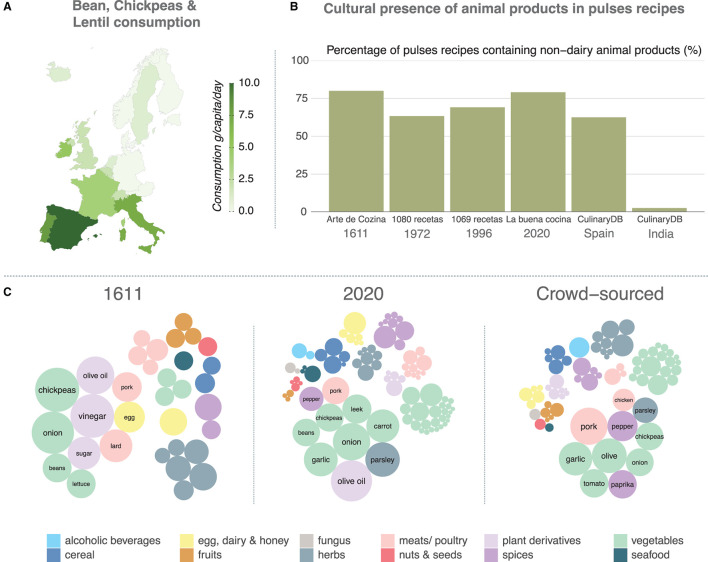
A complex systems view of food consumption. **(A)** shows a comparison of the intake of legumes in Southern, Western and Northern Europe, which reveals the role of sociocultural factors. Looking at the ingredients that are commonly paired with these legumes in Spanish cuisine, one finds that this consumption is heavily related to the consumption of non-dairy animal products, although this is not true for the Indian cuisine sample **(B)**. In **(C)**, each circle represents a single ingredient used in at least one recipe containing beans, chickpeas or lentils. The size of the circle is proportional to the number of recipes containing the ingredient and the color the group they belong to. In all cases only the top 10 ingredients are labeled.

To illustrate the hardships to be faced in the field of healthy nutrition, let us discuss one of the conclusions of the EAT-Lancet commission (32), namely, to have legumes as the main source of proteins, while red meat consumption should be minimal. Whereas data on risk of low intakes of red meat are imprecise, a diet low in legumes has been associated with a higher mortality rate (33). However, dietary recommendations for individual food items are often based on individual studies, without being supported by a comprehensive understanding of the interaction between foods and without fully accounting for the effects of substituted foods. It is therefore risky to translate independent observations into recommendations without further analysis. A complex system-wide perspective would reveal hidden correlations which in turn depend on multiple confounding factors such as cultural habits and tradition. Take as an example the consumption of beans, chickpeas and lentils in Western and Northern Europe in comparison to other regions of the continent with data from 2018 (34), see Figure 4A. This simple analysis already reveals important sociocultural differences that could undermine the success of the aforementioned recommendation by the EAT-Lancet commission. On the other hand, food raw materials are usually paired together (see Figure 1), therefore, the proposed solution might not be as simple as it appears. Although intake questionnaires have the potential to consider the pairing of multiple food products, these are rarely considered comprehensively when analyzing their relation to health.

For illustrative purposes, we have chosen a longitudinal set of recipe sources that, even though they cannot be directly employed as proxies of the actual recipes used by the population, at least they might let us uncover some patterns. In particular, we have first selected four recipe books containing traditional Spanish food. The first one is “Arte de Cozina,” a book published in 1611, which stands out by not having any references to (nowadays) common foodstuffs coming from America, such as tomatoes or potatoes. The second is “1080 recetas”, published in 1972 by Simone Ortega, whose French origin influenced many recipes in the book. Nonetheless, her book is still being reedited today and has sold over 3 million copies, and thus its influence on the recipes commonly cooked in Spanish households might be important. Lastly, we have selected two books written by Karlos Arguiñano, a famous Spanish chef who has hosted a cooking show on TV for more than 30 years and published more than 40 recipe books. As we can see in Figure 4B, in all these cases the percentage of pulses recipes containing non-dairy animal products is larger than 60%, reaching even 75% in the last book considered. Interestingly, the distribution is fairly constant throughout the books, signaling that, indeed, pairing animal products with legumes can be considered part of Spanish food culture. We have further explored an online database of parsed recipes extracted from several websites, CulinaryDB (35). Even though crowd-sourced recipes, such as the ones found online, can have many biases, we once again find a strong association between legumes and animal products in Spanish cuisine. On the other hand, if we look at recipes from India obtained in the same database, we find that this association is not present at all (Figure 4B).

Lastly, we explore the composition of recipes containing the aforementioned legumes in the recipes appearing in the book published in 1611, the one published in 2020 and online (Figure 4C). The first observation is that the number of ingredients increases significantly over time. It is also interesting to note how vinegar and lard disappear from modern cuisine. In particular, lard can only be found in one recipe over the whole set of recipes in the book from 2020, while it played a relevant role in 1611. This contrasts with central European countries in which animal fat is still one of the main sources of fat (36). On the other extreme, and related to the problems that we described previously in regard to quality and heterogeneity of data sources, we can see that recipes obtained from crowd-sourced data also differ from the ones found in printed sources. Olive oil, which is central to Spanish cuisine, does not appear in the online database and is substituted by the ingredient olive (Figure 4C). This reflects another challenge of analyzing large amounts of information, namely, disambiguating the terminology. Raw olives have many different properties, for example as a source of bioactives or as a ratio bioactive/fat, and are consumed in completely different recipes than their oil, and yet during the data cleaning process one might decide to consider them to be the same. A similar problem might appear in other foodstuffs, especially when they are regional or just a seemingly slight variation of the raw product.

The previous analyses, though simple, demonstrate the complexity behind cultural habits and their relation to nutrition. Given the emerging overall picture coming out from the above examples, it becomes clear that shifting from traditional diets to new, more sustainable diets is not straightforward. Finding and handling the proper data on food pairing and consumption in the population is key, but it does not represent the only open challenge. Accessing health data at a population level is relatively simple for diseases that are uniformly classified and registered. However, it remains difficult to identify disease risk factors, especially the metabolic ones, based on freely accessible data, even more when the evidence of causal associations between food consumption and health markers is not conclusive. The linkage between health, food consumption and other databases (e.g., personal care product consumption) at the individual level is also crucial for developing precision nutrition (37), studying multiple interactions and establishing causation, but are rarely available or allowed due to data protection. Finally, it is worth mentioning that an ecological approach could also be beneficial in terms of exploiting heterogeneities and differences of the populations, which might help expand the ability to identify associations. We hope that adopting a holistic perspective will increase our chances to move toward more healthy diets.

## Characterizing Causation

Before concluding, we also stress that an important issue and a crucial ingredient for the paradigm shift described here is to characterize causation, which should be of fundamental priority. Even intuitively, causation requires not just correlation, but counterfactual dependence. Therefore, it can only be inferred with some probability, never exactly known. However, a large part of the literature on data analytics, including that related to nutrition, is based on the principle that one can legitimately deduce a cause-effect relationship between two events solely based on an observed mutual correlation between them. In most cases, the latter does not hold. Thus, a paradigm shift on policy regarding the way we draw conclusions that lead to recommendations on healthy nutrition is also required. To this end, one can capitalize on statistical methods for data experiments that approximate at best the counterfactual state of the system. In particular, Bayesian statistics, based as it is on the Bayesian interpretation/expression of probability as a “degree of belief” in the happening of an event, may come to the rescue. The Bayesian method is grounded in Bayes' theorem, which describes the probability of an event based on prior knowledge in terms of conditional probabilities, so as to be able to compute and update probabilities after acquiring new data. This is not an easy task, as the formulation of mathematical models in terms of Bayesian statistics requires the specification a priori of probability distributions for the model parameters. Moreover, as the parameters of prior distributions may themselves have prior distributions, one has indeed a hierarchy, a network of Bayesian models. On the other hand, finding correlations does not make forecasting easier. Indeed, when forecasts are at stake, it is the Granger approach that enters the game. Granger's causality test aims at determining whether a given time series is useful in forecasting another. While ordinarily regressions reflect mere correlations, Granger's method assumes that causality may be tested for by measuring the ensuing ability to predict the future values of a dataset from the prior. Of course, what Granger test finds—and efficiently—is predictive causality, not true causality, as it identifies the cause-effect relations only as conserved co-occurrence. Furthermore, it can lead to wrong conclusions if confounding factors are uncontrolled, something that is especially hard to do—or even impossible—in any observational study. Rigorously, inference is instead essentially inductive reasoning, and a priori we should accept our inability to legitimately deduce a complete cause-effect relationship: that's why assuming that correlation implies causation is a logical fallacy. All of this is a severe collection of constraints on the quality, homogeneity and reliability of the datasets utilized. Notwithstanding, these statistical techniques can be complemented, or even superseded in some cases, by omics approaches and more direct, experimental techniques such as the ones based on biomarkers (38).

In summary, we believe that it is becoming apparent that existing guidelines and parameters defining healthy nutrition are not producing the desired results given the ever-increasing prevalence of food-related non-communicable diseases such as diabetes and obesity. There could be many reasons for this failure, including that recommendations are not backed up by data and the limitations inherent to the traditional approaches. This calls for the need to change the prevalent methodological approach. Here, we have discussed through a few examples, what new insights can be gained with such a toolbox. Our analyses suggest a roadmap that should include at least two areas of work: (i) the need to gather, mine and curate better food composition data, because with the current data we cannot go from specific to general recommendations—as of now, it is even impossible to have unique chemical composition of common foodstuff—, and (ii) the need of adopting radically new points of view and methodologies that account for many existing interdependencies in chemicals, nutrients and foods and their total and relative intake so as to advance in our quest to find new correlations and causation and in the redefinition of the parameters of healthy and sustainable nutrition. While we focused here on the consumption aspects of nutrition, we recognize that the sustainability of production is an additional societal concern, analysis of which ultimately needs to be incorporated in complete food chains. We believe that the foundations of such a roadmap is at the crossroad of Big Data, Artificial Intelligence and Complex Systems sciences.

## Data Availability Statement

The original contributions presented in the study are included in the article/supplementary material, further inquiries can be directed to the corresponding author/s.

## Author Contributions

YM and AA wrote the first draft of the manuscript. FB, OJ, EM, RS, and MR wrote sections of the manuscript. All authors contributed to conception and design of the study. All authors contributed to manuscript revision, read, and approved the submitted version.

## Funding

The Sustainable Nutrition Scientific Board research was carried out independently thanks to the financial support of Soremartec SA and Soremartec Italia, Ferrero Group. The funders had no role in study design, data collection, and analysis, decision to publish, or preparation of the manuscript.

## Conflict of Interest

EM is employed by Borneo Futures, co-chair of the IUCN Oil Crops Task Force and has conducted work for the Roundtable on Sustainable Palm Oil and palm oil companies. OJ, EM, RS, and MR are independent members of the Sustainable Nutrition Scientific Board. The remaining authors declare that the research was conducted in the absence of any commercial or financial relationships that could be construed as a potential conflict of interest.

## Publisher's Note

All claims expressed in this article are solely those of the authors and do not necessarily represent those of their affiliated organizations, or those of the publisher, the editors and the reviewers. Any product that may be evaluated in this article, or claim that may be made by its manufacturer, is not guaranteed or endorsed by the publisher.
